# Research on university profiles about entrepreneurship and innovation orientation: Case of a developing country

**DOI:** 10.3389/fpsyg.2022.968996

**Published:** 2022-11-22

**Authors:** Umut Akcil, Dinara Suhanberdyyeva

**Affiliations:** ^1^Department of Educational Sciences, Near East University, Nicosia, Cyprus; ^2^Department of Educational Administration and Supervision, Near East University, Nicosia, Cyprus

**Keywords:** entrepreneurship, innovation, developing countries, universities, leadership in education

## Introduction

Higher education plays an important role in the socio-economic improvement in the world. Higher education institutions are generally perceived to be organizations that assume the responsibility of meeting the society's manpower needs and helping to solve the problems of the society through scientific research (Baskan, 2001). Higher education institutions have evolved in parallel to the social changes and kept up with the times since they were first established. All forms of educational organizations develop as a result of the industrial revolutions of their era. Until the middle of the nineteenth century, the mission of universities was education and teaching (Jencks, 1968); however, it is seen that in the third and even the fourth revolution phases, their mission has become to industrialize education-teaching and research activities and contribute to the society with positive outputs (Yildiz, 2019).

The third generation universities have begun to be discussed since the revolution period began at the start of the twentieth century. It is clear that the main cause of this is the rapid development of technology and globalization. Technology has become a leading target tool in responding to the changing needs of education (Korkmaz and Mirici, 2021). In the light of these developments, we closely follow the implications that the digital age parallel to technological developments has on the education sector (Akcil and Baştaş, 2020). In this process in which the ever-increasing globalization and international competition have accelerated, the developing “university-industry cooperation” activities have led to the formation of entrepreneurial universities. Universities in the information economy approach attach more importance to entrepreneurship, shape their faculties accordingly and industrialize the results of their research. New technologies, new types of students, new higher education providers, profit-seeking corporate universities, virtual universities, globalizing students and the expectations of the job market are at the forefront in the changes occurring in universities (Scott, 2011; Erdem, 2016). It is seen that universities have played a pioneering role in changing the society within the fourth revolution phase since the first quarter of the twenty-first century. It has become necessary for the universities to help develop the society in which they are in and for the society to collaborate with the universities. It is seen that the two important factors of “thematic” and “transformative” in this phase are reflected in the missions of universities. In fourth generation universities, different disciplines work together in more flexible manner and interdisciplinary limitations are reduced (Erdem, 2016).

As a result of the third and fourth revolutions, in other words, in today's industrial revolution, the competitive structure of the world has turned universities into entrepreneurial and innovative organizations that are interlinked with industry, have their own patents, can earn an income from their own centers and institutes, and have the criteria of R&D, innovation, production and competitiveness in the transformation and development phase.

It is clear that the conceptual definitions of entrepreneurial universities are affected by this evolutional phase. In this context, it is expected that entrepreneurial universities are institutions that are socio-entrepreneurial and innovative across different time periods; capable of carrying out education-teaching, research and social development activities together; combining the objectives of economic, social development, innovation, knowledge production, providing innovative benefits to the society and technology transfer in their mission statement; have features such as quality research and development activities and an ecosystem that will provide new products and services (Etzkowitz et al., 2000; Etzkowitz, 2003; Montesinos, 2008; Mets, 2010; Walker, 2012). Clark (2001), who examined the topic of entrepreneurial universities in Europe, argued for the necessity of integrating the entrepreneurship culture into universities by maintaining the balance between academic culture and market culture and a corresponding transformation in addition to education-training and scientific research (Yamamoto, 2020).

Another important aspect of entrepreneurial universities is that they are innovation focused. Therefore, entrepreneurial universities form their corporate strategies around innovation because the path to innovation lies in being entrepreneurial (Schumpeter et al., 2017).

In the light of the needs specified above, it can be said that the new generation higher education institutions should be open to the concepts of entrepreneurship and innovation to remain up to date with current developments. In this framework, the entrepreneurship and innovation terms can be explained as below.

### Theoretical framework

The term entrepreneurship first began to be scientifically examined in the work of Joseph Schumpeter. According to Schumpeter, if the entrepreneur is only innovating then they are an entrepreneur (Odabaşi, 2007).

In the 1990's the entrepreneurship university model emerged in universities in the United States and it has now spread throughout European universities (Sakinç and Bursalioglu, 2012). In this century, we often observe Entrepreneurial and Innovative university terminology. It originates from the French verb “entreprendre” which means “to do something different” (Odabaşi, 2007). An entrepreneurial university is a university that can form innovative views, add value to a society, and can be different and develop. The terms entrepreneurial and innovative are often interlinked. Innovation is the most important aspect of entrepreneurialism and entrepreneurs play an important role in feeding innovation because innovation is one of the sources of entrepreneurship and innovation can be defined as a university that enables entrepreneurs to present a different work or service and that can carry out interdisciplinary studies and collaborations (Ömürbek and Karataş, 2018).

This new university model has become a group of organizations that take on the role of teaching, research and public service with the aim of meeting the needs of the external world and the demands of the market. This new organization is called an entrepreneurial university and its research and education content have changed. Today, they do not just do “science for the sake of science,” but they do teaching and research to solve practical problems. Universities that follow this model evaluate the potential of the regions in which they are located and contribute to the region through research and development (Arap, 2010; Çiftçi, 2010). Consequently, it is important for development for the university to examine the region in which it is located or on a wider global scale such that it can add value. In this regard, it is possible to discuss certain models than can be used to measure both entrepreneurialism and innovation.

Experts in the field of entrepreneurialism stress the importance of studies that measure university entrepreneurialism and refer to models where certain criteria are used. These models, which are used by developing countries, help develop the entrepreneurialism, innovation and creativity of universities, academicians, students, workers and researchers and contribute to the development of society. In this framework, Robertson (2008) summarized the key aspects of entrepreneurial universities as follows:

- A strong leadership that develops the entrepreneurial capacity of all university actors on the campus,
- Establishing strong ties with external stakeholders that create added value, increasing university-industry cooperation,
- Ensuring entrepreneurial results that have an effect on individuals and institutions,
- Applying innovative learning techniques that result in entrepreneurial behavior, removing limitations to support effective information flow between institutions,
- Multidisciplinary education approaches that focuses on solutions for complex world problems and that reflect real world experience,
- Entrepreneurial thought and encouraging leadership applications.

Similarly, it is possible to discuss similar criteria with regard to the innovation concept. Reuters (2022) focused on universities obtaining patents, which is used as a criteria in the innovative university rankings. Additionally, the other criteria can be listed as follows:

- Number of patents,
- Success of patents,
- Patent Citation Index,
- Patent Citation Impact,
- Patent Percentage which is References,
- Industrial Impact,
- Projects that have co-authors with industry,
- The total number of articles published by the university.

The interest toward entrepreneurial and innovative university models is continually increasing among institutions and organizations that have higher education policies. As in the rest of the world, in recent years, similar models have been developed in Turkey. The Scientific and Technological Research Council of Turkey (Tubitak) has an entrepreneurship and innovation index model in which universities are regularly evaluated every year (Tubitak, 2013). The specified dimensions according to this index are as follows:

- Scientific and Technological Research Competency
- Intellectual Property Pool
- Collaboration and Interaction
- Entrepreneurship and Innovation Culture
- Economic Contributions and Commercialisation

Indicators under the main dimensions in the evaluation model determined by Tubitak are explained in detail in the methodology section of this research. When these are examined, it will be seen that the content is similar to that of international organizations such as Reuters. This research has been designed within the framework of the Tubitak entrepreneurship and innovation index model, since the working group included in this research and the higher education institutions in Turkey have connections in terms of the language of education, education system and collaboration.

## The aim of the study

According to the innovative university listings on Reuters (2022), it is seen that most of the entrepreneurial and innovative universities in the world are from developed countries. It is believed that the situation in less developed or developing countries must also be investigated. Therefore, a vision can be specified for the development of higher education institutions in underdeveloped countries and recommendations to raise them to the level of developed countries will be developed.

Moving forward from this point, this research was carried out with a sample from Northern Cyprus, as it is an underdeveloped country due to its lack of international recognition. It is aimed to investigate the entrepreneurship and innovation orientation of universities by examining the strategic plan documents of higher education institutions and with the scale in which the entrepreneurship and innovation models mentioned in the theoretical framework of the research are used. The research questions developed to achieve this aim are as follows:

1. What are the entrepreneurial and innovative levels of universities from the perspective of academicians?
2. Based on the document study, what are the entrepreneurial and innovation levels of universities?

### Methodology

The mixed research model was used in accordance with the general aim of this study. An enriched design where the quantitative and qualitative data were obtained and analyzed simultaneously was used (Tashakkori and Teddlie, 2010). Creswell (2021) stated that mixed method research helps answer questions that quantitative or qualitative research method cannot answer alone and that it approaches situations and cases from a more holistic view.

The survey model was used in the quantitative stage of this study. Survey research is based on the opinions of the participants about a subject or event, or their interests, skills, abilities, attitudes, etc. It is a type of research in which the characteristics of the research are determined and the situation in the past is tried to be revealed as it is, and relatively large samples are studied (Karasar, 2005, s. 77; Brewer and Wang, 2015). In this study, the oldest and largest five universities in the country were accessed and the academician's views were collected using the survey model.

The document analysis model was used in the qualitative phase of this study. This technique is one of the qualitative research methods. It is a technique used to collect first hand data through analyzing the content of materials in research where observation or meeting in person are not possible (Yildirim and Simşek, 2016). The information in the documents can show certain questions that must be asked and the present situation. Documents are a tool that help to analyse change and development (Bowen, 2009). The documents on the official web pages and additional documents obtained from the five largest universities were used in this research.

### Sample group

The sample group for the quantitative section of this study was selected using a simple random sampling method. The accessible population of the research is 2,500 people. Considering 95% reliability and 5% margin of error and taking into account the frequency of opinions to be examined, it was aimed to include 330 participants. Although this number was achieved, data that were determined to be incorrect and missing were removed, making the total number of participants 224. Three experts were asked and he said that the data collected from the sample could be used, provided that the data met the normal distribution. All of the participants were working as teaching staff at the five largest universities (2 State-supported, 3 Private universities) in the North of Cyprus in the 2020–21 academic year. When the age distribution of the participants is examined, it is seen that 75% were between the ages of 35–44 and 65% were between the ages of 65–74. When the distribution of participants amongst the faculties is examined, it is seen that the largest percentage of 40% were from the Health Sciences Faculty and the lowest percentage of 4% were from the Agricultural Sciences Faculty. When the distribution across private and state universities is examined, it is found that 84.4% of the lecturers were working at private universities and 15.2% were working at state universities. For further info you can check data set available: https://doi.org/10.5281/zenodo.6642028.

In the qualitative part of the research, the strategic plan documents found on the official websites of the five major universities in the 2020–2021 academic year and the strategic plan documents received from the Ministry of National Education through official correspondence were studied. The documents subjected to document analysis were selected with the sampling method. Comprehensive and selective documents are important for a holistic evaluation (Hodder, 2000).

### Data collection process

In the quantitative part of the research, a 5-point Likert scale (“strongly agree,” “agree,” “somewhat agree,” “disagree,” and “strongly disagree”), and a scale that determines the innovation and entrepreneurship level of institutions were used. The scale was created by Semra Bayrakçi and the previous reliability coefficient was found to be α: 0.97 (Cronbach's alpha). Opinions were obtained from three experts (one research design lecturer: Assoc. Prof.; two field experts: Prof.) regarding the usability of this scale on lecturers working at universities in our country. Based on the views of the experts, it was decided to use the scale in its current state as scope validity was confirmed. The reliability coefficient for the scale being applied in Northern Cyprus was determined as α: 0.98 (Cronbach's alpha). The scale includes a total of 42 items and sub-dimensions. The sub-dimensions include; Leadership Innovation, Manager Operation Innovation, Student Counseling and Activity Innovation, Curriculum and Teaching Innovation, Innovation of Lecturers in Specialism Development, Resource Application Innovation, and Campus Building (Architectural) Innovation. The researchers collaborated with the Higher Education Department of the Ministry of National Education in the country for the application of the scales. Permission was obtained from the Ministry to visit the five largest universities and collect data. The scales were created electronically using Google Forms. The Google Form links were shared with the rectors of the universities. The quantitative data were collected in the fall and spring semesters of the 2021–22 academic year over a period of 5 months.

In the qualitative part of the research, five public and accessible documents on the official web pages of the 2 state and 3 private universities were examined for document analysis. This process was carried out simultaneously with the qualitative data collection process and took ~3 months. The following procedure was carried out with the documents obtained: (1) the documents were accessed and downloaded onto the computer of the researcher, (2) their originality was checked and, (3) the documents were selected. The next phase is related to the analysis process and is explained in detail under the next heading.

### Data analyses process

The quantitative answers of 224 participants who participated voluntarily in the quantitative dimension of the study were collected using the Google Form. This information was then transferred to the Excel program. Then, this set of data was uploaded onto the SPSS 24.0 program. Frequency analysis was used to determine whether or not there were any missing or incorrect data. Following this, the normal distribution of the quantitative data and the reliability coefficient of the collected data (Cronbach alpha “α”) were examined. Due to a normal distribution being obtained according to the statistical information seen in Table 3 (skewness and kurtosis −1 - +1; mean ≅ median) it was decided to make use of parametric tests. Frequency analysis was used for the distribution of personal information, and total score calculation and arithmetic average analysis were used to reveal the innovativeness and entrepreneurship levels of the institutions. The *t*-test was used to analyse the difference between the innovativeness and entrepreneurship levels of private and public universities, which is the independent variable, and ANOVA analyses were used to examine whether there was a difference between innovation and entrepreneurship scores according to science departments. The *t*-test is used to test the statistical significance of the difference between the two averages. The Anova test is a tool used to measure if there is a statistically meaningful difference between the averages of more than two independent groups (Kaur and Kumar, 2015). The significance level in this analysis was determined to be (p) 0.05. In the case where it is significant, Scheffe analysis was used for multiple comparisons. This test assumes the equality of the variances, but does not consider the assumption that the number of observations in the group have to be equal (Büyüköztürk, 2007). For further info you can check data set available: https://doi.org/10.5281/zenodo.6642028.

Descriptive statistics were used for the analysis of qualitative data. The document analysis was used to systematically analyse the content of the written documents (Wach and Ward, 2013). The descriptive approach tries to reveal that which exists through the question “what was found?” and ensures the data are collected and analyzed according to the previously determined framework and themes (Wolcott, 1994). In the light of this information, it was decided that the most suitable technique to be used for the analysis of documents was the descriptive technique. According to this technique: (1) While creating a framework for descriptive analysis, the entrepreneurship and innovation index evaluation criteria determined by Tubitak (2013) were used; (2) According to the criteria specified in the index, give themes with the titles “Scientific and Technological Research Competency, Intellectual Property Pool, Collaboration and Interaction, Entrepreneurship and Innovation Culture, Economic Contributions and Commercialisation” were used (3) In line with the themes, the documents were searched according to the sub-indicators; (4) the findings were inserted into tables and discussed. The information that was inserted into the tables was presented as frequency and percentage. Based on the indicators of the theme and index used in the document analysis, the codes found in the documents can be seen at: For further info you can check data set available: https://doi.org/10.5281/zenodo.6642028.

### Validity and reliability analysis

It is important to conduct reliability and validity analysis for both quantitative and qualitative research (Yildirim and Simşek, 2016). Statistical procedures were carried out using the SPSS program for the validity and reliability of the data collected in the qualitative research. In the qualitative research, a “credibility” study was conducted for this process. Lincoln and Guba (1985) drew attention to the fact that trustworthiness is more necessary than validity and reliability in qualitative research. The best way to ensure trustworthiness in a qualitative study is long-term interaction. In this study, the researcher interacted with the documents for a long period of time. The researcher examined the documents in an unbiased manner and placed the information in tables under different themes. The information obtained from the documents were given under the tables with the “direct quotation” method and discussed. The second researcher also observed and confirmed this process. Additionally, the document analysis procedure is discussed in detail in the methodology section. Therefore, the “credibility” process recommended by Lincoln and Guba was carried out.

## Results

In the framework of the first sub-aim of the research, the answer to the question “1-What are the entrepreneurial and innovative levels of universities from the viewpoints of academicians?” was sought. The SPSS program was used for analysis to answer this question. The results of the analysis conducted in the order of arithmetic mean and total score calculation test, *t*-test, and Anova tests are given in Tables 1, 2.

**Table 1:** The arithmetic mean of the whole scale and its sub-dimensions, and the total score distribution of the faculty units.

**Factor**	***N* (items)**	** $\bar{\text{x}}$ **
In the field of leadership	6	3.71
In the field of administrative operations	8	3.54
In the field of student counseling and activities	5	3.40
In the field of curriculum and instruction	7	3.78
In the field of specializations of teaching staff	5	3.96
In the resources and applications area	6	3.48
In the field of campus structure (architecture)	5	3.79
Total point	42	3.63
**Faculty departments**	***N*** **(participants)**	$\bar{\text{x}}$
(1) Faculty of economics and administrative sciences	29	149.55
(2) Faculty of education	31	169.67
(3) Faculty of arts and sciences	8	142.37
(4) Faculty of engineering	21	145.95
(5) Civil and environmental engineering	5	150.20
(6) Faculty of medicine	6	162.00
(7) Faculty of law	6	139.33
(8) Faculty of architecture	25	162.72
(9) Faculty of tourism	10	171.70
(10) Faculty of health sciences	40	149.02
(11) Faculty of communication	10	157.10
(12) Faculty of sports sciences	15	122.00
(13) Faculty of pharmacy	6	157.50
(14) Faculty of dentistry	4	162.00
(15) Faculty of agricultural sciences and technologies	4	197.75
(16) School of computer and technology	4	139.25
Total	224	153.78
State university total	34	155.58
Private university total	190	153.46

**Table 2:** Innovation and entrepreneurialism difference between the units of the universities Anova test.

	**Sum of squares**	**df**	**Mean square**	***F***	***p***	**Difference**
Between groups	42,706,792	15	2,847,119	2,516	0.002	*p* < 0. 05
Within groups	235,330,922	208	1,131,399			12&2; 12&8; 12&9; 12&15
Total	278,037,714	223				

Levene statistic df1: 4, df2: 219; p: 0.267 / p: 0.05.

When Table 1 is examined, it is seen that the entrepreneurial and innovation levels of the five oldest universities are at the “I agree” level (x = 3.63). According to this finding, it can be said that the universities have an entrepreneurial and innovation approach. Additionally, after a comparative analysis made between the faculty units of the universities, as can be seen in Table 2, no significant differences were seen between the Sports Sciences, Education, Architecture, Tourism and Agriculture Faculties (*p* = 0.05). According to this finding, the faculties that have the highest level of entrepreneurship and innovation are respectively the Agricultural Sciences Faculty (x = 197.75), Tourism Faculty (x = 171.70), and Education Faculty (x = 169.67). It is seen that the faculty with the lowest level is the Sports Sciences Faculty. The reason for these findings could be that the country's two most important sources of livelihood are Tourism and Agriculture and Livestock. Although the findings in Table 1 show that the entrepreneurial and innovation levels of the universities are high, the document analysis in the second sub-aim of the study shows that the current situation is not good.

The second sub-aim of the study, searched for an answer to the question “2-Based on the document study, what are the entrepreneurial and innovation levels of the universities?” The documents were examined with the descriptive analysis technique. The findings obtained are presented in can be seen at: For further info you can check data set available: https://doi.org/10.5281/zenodo.6642028.

When the outcomes of the descriptive document analysis process for the strategic plans of the universities in Table 5 are examined, according to the themes of the entrepreneur and innovation index, it is seen that the most information is in the dimension of “Scientific and Technological Research Competence” (+). It is seen that the second most information is in the “collaboration and interaction” dimension. No information could be found in the strategic plans in the dimensions of “Intellectual Property Pool” and “Economic Contribution and Commercialization.” According to this finding, it is seen that the academician and academic activities of academic publications, citations, thesis, projects, and graduation are more at the forefront. It is very interesting that there is no information on economic contribution, commercialization and intellectual property. In the light of these findings, it can be said that there are no targets, studies, practices and discourses on entrepreneurship and innovation in the strategic plans that reflect the current situation and future plans of the relevant universities.

## Discussion

Research on this topic has not previously been carried out in a developing or a non-developed country. The current situation in countries that are not in the Reuters listings, which largely includes developed countries, has not been researched. It is believed that a current situation analysis should be performed in order to create a roadmap for how developing countries and their universities can develop with such research. Entrepreneurialism and related concepts constitute one of the most important mechanisms of economic development (Bunyasrie, 2010). Higher education institutions act as an important catalyst for the development of a country (Sakinç and Bursalioglu, 2012). In order to close the education quality gap between developed and developing countries, it is necessary to contribute to adequate education and training at higher education level (Vo, 2022).

It was an expected result that the academicians working at the universities would exhibit biased behavior. Due to the fact that this research was carried out with a parallel and equally dominant mixed method approach, after the analysis of the strategic plans (qualitative data) of the universities selected for the study group in the qualitative part of the research, quantitative data and qualitative data were evaluated from different perspectives.

When the results according to the academicians' views were examined, the universities' entrepreneurial and innovation levels were found to be high in the areas of thesis, lecturer specializations, curriculum, student relations, and education. As a result of the analysis of the strategic plans, it can be said that this is consistent with the findings related to the theme of “Scientific and Technological Research Competence” in which academicians play a more active role. The result that these institutions were weak in other themes was concluded. Higher education institutions, which combine the objectives of economic and social development, innovative knowledge production, providing innovative benefits to the society and technology transfer, are considered to be institutions that have accepted the mission of being entrepreneurial universities (Mets, 2010). As can be understood from this explanation, quantitative research and number of publications alone are not enough to be an entrepreneurial and innovative university.

Again, according to the results of the analysis of the strategic plans, the fact that no data were available for the “Economic Contribution and Commercialisation” and “Intellectual Property Development” themes is a deficiency in terms of the objective of becoming an innovative and entrepreneurial university. It is important for scientific research to add value to the society, social capital and corporate structure. Higher education institutions that carry out education, teaching, research, economic and social development activities together transform the theoretical and practical knowledge they have into economic values and add value to the community, region and country they are in must be created (Etzkowitz, 2003).

Furthermore, the results obtained from the “Collaboration and Interaction” theme used in the strategic plan analysis are interesting. According to these results, it is seen that there is no collaboration between industry and the universities, the universities do not obtain funding from industry and that the organizations do not have such an aim. According to the research and the reports of international organizations, it is not sufficient to only produce academic products as collaborations must be formed between job sectors and entrepreneurial activities must be carried out (Wachter et al., 2015). Again, in another research, it was emphasized that key aspect of entrepreneurial universities is industry, state and university collaboration (Siegel and Link, 2003).

Entrepreneurial universities must have a social capital with an innovation focussed and entrepreneurial spirit and an entrepreneurial culture formed by the management, personnel and students (Yildiz, 2019). In this research, it should be noted that there are very few indicators under the theme of “entrepreneurship and innovation culture” in the strategic plans.

Another result obtained from the data obtained from the academicians was related to the entrepreneurial and innovation levels of the faculties. At this point, it was concluded that the entrepreneurial and innovation levels were higher in the “Tourism and Agriculture” faculties compared to other faculties. It can be said that this result is closely connected to the reality of the country as the main economic source of the north of Cyprus is from these two sectors. It is also an important problem that the impact of the industrial sector in this country on development has remained in the 10% band for years (Sansal, 2007). When these facts are put aside, it must be stated that other fields are also important for the development of industrialization and innovation culture in universities. In particularly, they must conduct activities in the field of science and engineering, perform high quality scientific research and establish techno parks to carry out research and development activities (Link and Scott, 2007; Albahari, 2019).

It is known that stagnation in industry and production is at the root of the problems experienced in every developing country. It can be said that it is possible to solve this problem through industry-university and state-university collaborations and support, national and international funds, new ideas and patents. Encouraging scientific discoveries, ensuring the support of industry and the state for universities to conduct research and produce innovative products will encourage entrepreneurial activities on university campuses (Roach, 2017). Thus, as universities develop in the current era, the development of countries can be ensured with the increase in the level of entrepreneurship. Entrepreneurship is one of the most important mechanisms of development.

## Data availability statement

The original contributions presented in the study are included in the article/supplementary materials, further inquiries can be directed to the corresponding author/s.

## Ethics statement

The studies involving human participants were reviewed and approved by Ethical Committee Board of Near East University. The patients/participants provided their written informed consent to participate in this study.

## Author contributions

UA wrote the article, did the conceptualization of it, and contributed to the methodology of the research. DS contributed to the generation of the dataset and helped to the validation of the study. All authors contributed to the article and approved the submitted version.

## Conflict of interest

The authors declare that the research was conducted in the absence of any commercial or financial relationships that could be construed as a potential conflict of interest.

## Publisher's note

All claims expressed in this article are solely those of the authors and do not necessarily represent those of their affiliated organizations, or those of the publisher, the editors and the reviewers. Any product that may be evaluated in this article, or claim that may be made by its manufacturer, is not guaranteed or endorsed by the publisher.
